# Guidelines on models of diabetic heart disease

**DOI:** 10.1152/ajpheart.00058.2022

**Published:** 2022-06-03

**Authors:** Lisa C. Heather, Anne D. Hafstad, Ganesh V. Halade, Romain Harmancey, Kimberley M. Mellor, Paras K. Mishra, Erin E. Mulvihill, Miranda Nabben, Michinari Nakamura, Oliver J. Rider, Matthieu Ruiz, Adam R. Wende, John R. Ussher

**Affiliations:** ^1^Department of Physiology, Anatomy and Genetics, University of Oxford, Oxford, United Kingdom; ^2^Department of Medical Biology, Faculty of Health Sciences, UiT—The Arctic University of Norway, Tromso, Norway; ^3^Department of Medicine, The University of Alabama at Birmingham, Birmingham, Alabama; ^4^Division of Cardiology, Department of Internal Medicine, McGovern Medical School, The University of Texas Health Science Center at Houston, Houston, Texas; ^5^Department of Physiology, University of Auckland, Auckland, New Zealand; ^6^Department of Cellular and Integrative Physiology, University of Nebraska Medical Center, Omaha, Nebraska; ^7^Energy Substrate Laboratory, University of Ottawa Heart Institute, Ottawa, Ontario, Canada; ^8^Department of Biochemistry, Microbiology and Immunology, University of Ottawa, Ottawa, Ontario, Canada; ^9^Department of Genetics and Cell Biology, Maastricht University Medical Center, CARIM School of Cardiovascular Diseases, Maastricht, The Netherlands; ^10^Department of Clinical Genetics, Maastricht University Medical Center, CARIM School of Cardiovascular Diseases, Maastricht, The Netherlands; ^11^Department of Cell Biology and Molecular Medicine, Cardiovascular Research Institute, Rutgers New Jersey Medical School, Newark, New Jersey; ^12^Radcliffe Department of Medicine, University of Oxford Centre for Clinical Magnetic Resonance Research, University of Oxford, Oxford, United Kingdom; ^13^Montreal Heart Institute, Montreal, Quebec, Canada; ^14^Department of Nutrition, Université de Montréal, Montreal, Quebec, Canada; ^15^Department of Pathology, University of Alabama at Birmingham, Birmingham, Alabama; ^16^Faculty of Pharmacy and Pharmaceutical Sciences, University of Alberta, Edmonton, Alberta, Canada; ^17^Alberta Diabetes Institute, University of Alberta, Edmonton, Alberta, Canada; ^18^Mazankowski Alberta Heart Institute, University of Alberta, Edmonton, Alberta, Canada

**Keywords:** cardiac function, diabetic cardiomyopathy, obesity, type 1 diabetes, type 2 diabetes

## Abstract

Diabetes is a major risk factor for cardiovascular diseases, including diabetic cardiomyopathy, atherosclerosis, myocardial infarction, and heart failure. As cardiovascular disease represents the number one cause of death in people with diabetes, there has been a major emphasis on understanding the mechanisms by which diabetes promotes cardiovascular disease, and how antidiabetic therapies impact diabetic heart disease. With a wide array of models to study diabetes (both type 1 and type 2), the field has made major progress in answering these questions. However, each model has its own inherent limitations. Therefore, the purpose of this guidelines document is to provide the field with information on which aspects of cardiovascular disease in the human diabetic population are most accurately reproduced by the available models. This review aims to emphasize the advantages and disadvantages of each model, and to highlight the practical challenges and technical considerations involved. We will review the preclinical animal models of diabetes (based on their method of induction), appraise models of diabetes-related atherosclerosis and heart failure, and discuss in vitro models of diabetic heart disease. These guidelines will allow researchers to select the appropriate model of diabetic heart disease, depending on the specific research question being addressed.

## INTRODUCTION

Diabetes continues to increase at an alarming rate, with current estimates now indicating that there will be ∼700 million people worldwide living with diabetes by 2045 (1). Despite effective control of glycemia (targeting glycated hemoglobin <7.0%) being positively associated with reduced risk for microvascular complications (2), the majority of deaths in individuals with diabetes are due to macrovascular complications (3–5). Big data studies totaling over 1.9 million people showed that diabetes increases the risk of angina and myocardial infarction, with peripheral arterial disease and heart failure being the most common initial manifestations of cardiovascular disease in type 2 diabetes (T2D) (6). Therefore, there is currently an unmet need for cardiovascular therapies for patients with diabetes. Furthermore, major health regulatory agencies (e.g., US Food and Drug Administration and European Medicines Agency) have mandated that all new therapies in development for diabetes undergo rigorous assessment of cardiovascular risk through large-scale cardiovascular outcome trials before approval. This has resulted in a greater demand for better preclinical models of diabetes not only for the development of cardiac therapies but also for the early identification of deleterious cardiac side effects.

In the past 10–20 years, the field has made great strides in identifying key mechanisms driving diabetes-related heart disease (extensively reviewed in Refs. 4 and 7), which has been greatly aided by the development and improved characterization of models of diabetes, primarily in animals. The requirements of a model of diabetic heart disease depend upon the specific scientific question being asked, but broadly the model needs to replicate the human condition, replicate the mechanistic changes occurring within the heart of a person with diabetes, or replicate the drivers of diabetic myocardial dysfunction. The model must be reproducible, easily accessible, and fall within the remit of animal guidelines within the country of research. Although there is currently no specific model of diabetes whose associated cardiac dysfunction perfectly models the human disease, the purpose of this guidelines document is to provide the field with information on which aspects of cardiovascular disease are best represented by the available models. This review aims to emphasize the advantages and disadvantages of using these models to investigate mechanisms and potential treatments of cardiovascular diseases, and to highlight the practical challenges and technical considerations involved. We will herein review the preclinical models of diabetes according to their method of induction—dietary, pharmacological, and genetic—focusing first on T2D and then on type 1 diabetes (T1D). A discussion of models of diabetes-related atherosclerosis and heart failure is also included, as well as in vitro models of diabetes.

## THE CLINICAL PICTURE OF DIABETIC HEART DISEASE

Diabetes is a major risk factor for vascular disease including both microvascular (retinopathy, nephropathy, coronary microvascular, and neuropathy) and macrovascular manifestations (peripheral vascular disease, cerebrovascular, and coronary artery disease). Patients with diabetes have a two- to fourfold increased risk of coronary heart disease and ischemic stroke, and a 1.5- to 3.6-fold increase in mortality. As such, diabetes is a major risk for adverse cardiovascular events, and is such a powerful risk factor that it has been considered a “cardiovascular risk equivalent” (i.e., patients with diabetes but without coronary heart disease have a similar coronary mortality to patients without diabetes who had a previous coronary event) (8).

The term diabetic cardiomyopathy refers in the broadest sense to cardiac morphological and functional changes that occur because of diabetes, and importantly in the absence of other etiologies that exert their own independent effects, for example, independent of coronary artery disease, hypertension, valvular, or congenital heart disorders. One of the major challenges is the lack of a universally accepted and consistently applied definition of diabetic cardiomyopathy, with several definitions being used that cover the whole spectrum of diabetic heart disease from subclinical changes to overt heart failure. It is further complicated by the bidirectional link between diabetes and heart failure in humans, where diabetes increases the risk of heart failure, and heart failure itself increases the risk of T2D. With no universally accepted definition, it is difficult to assess the true incidence of diabetic cardiomyopathy.

Although they may vary in severity, it is now widely accepted that several common cardiac structural changes are seen in humans with diabetes. Left ventricular (LV) hypertrophy (defined for the purposes of this review as elevated total LV mass) is commonly seen in adults with diabetes, with around 70% showing some form of hypertrophy (9). Although both eccentric hypertrophy (elevated LV cavity size and preserved wall thickness) and concentric hypertrophy (normal or reduced cavity size and elevated wall thickness) are both reported, it is now generally accepted that reduced LV cavity size and concentric LV hypertrophy represent the main structural characteristics of diabetic heart disease (10). However, any cardiac hypertrophic remodeling must be taken in the context of the patients’ sex (11), ethnicity (12), body habitus (13), and arterial blood pressure (14). Patients with diabetes have evidence of diffuse myocardial fibrosis and expanded extracellular volume (15), as detected using magnetic resonance imaging (MRI) techniques.

Diastolic dysfunction is the earliest functional change in diabetic cardiomyopathy. Observational studies have found an increased frequency of diastolic dysfunction in T2D with prevalence varying from 20% to 78% depending on the criteria used (16, 17). Heart failure with preserved ejection fraction (HFpEF) is emerging as a major complication for patients with diabetes, with 30%–40% of patients with HFpEF also having diabetes (18). However, the mechanisms linking diabetes to this growing prevalence of HFpEF are currently undefined. There is a clear epidemiological relationship between T2D and heart failure with reduced ejection fraction (HFrEF), however, there are very few, if any, studies linking diabetes per se, in the absence of myocardial infarction, to progressive systolic decline. Most of the human studies reporting systolic dysfunction describe subclinical changes in systolic strain (12), rather than global changes in LV ejection fraction (LVEF).

Traditionally, cardiac dysfunction in diabetes is thought to progress along a spectrum from subclinical diastolic dysfunction to subclinical systolic dysfunction and then to overt systolic dysfunction and HFrEF. Although all are seen in diabetes, the evidence for this progression, in humans, is weak. Rather than being successive stages of diabetic cardiomyopathy, it is now thought that the HFrEF (dilated cavity and reduced systolic function) and HFpEF (reduced cavity size, concentric LV hypertrophy, and diastolic dysfunction) are not successive stages, but rather develop as separate phenotypes. The latest theories suggest that in the HFpEF phenotype, the central pathology is concentric LV remodeling resulting from coronary microvascular dysfunction, myocardial fibrosis, and metabolic dysregulation, whereas in HFrEF eccentric LV remodeling results from cardiomyocyte cell death, fibrosis, and microvascular rarefaction (19). What determines whether someone with diabetes develops HFpEF or HFrEF is unknown but is likely multifactorial and influenced by both genes and the environment.

Cardiac metabolic changes are almost universally reported in human studies and involve a metabolic shift away from utilization of glucose toward a greater utilization of fatty acids. Using positron emission tomography, diabetes has been shown to be associated with increased myocardial fatty acid utilization, increased oxygen consumption, and reduced cardiac efficiency (20, 21). When coupled with the evidence of reduced carbohydrate uptake and metabolism and the recent hyperpolarized ^13^C study showing reduced pyruvate dehydrogenase flux (21–23), this suggests a shift in cardiac energy substrate utilization from carbohydrate to lipids in humans. Although lipid accumulation is commonly reported in diabetes (24, 25) and is linked to diastolic dysfunction (26), the evidence for lipotoxicity as the causative mechanism is not well developed in humans. Similarly, glucotoxicity, as shown by changes in the receptor for advanced glycation end products, is associated with cardiovascular disease and mortality in people with diabetes. However, mechanistic studies showing a causative relationship are lacking (27, 28). With ^31^P magnetic resonance spectroscopy, a decreased myocardial phosphocreatine (PCr)-to-adenosine triphosphate (ATP) ratio has been described (23, 24), suggesting that myocardial energetic impairment is a key component in the pathophysiology of human diabetic heart disease. In addition, impaired metabolic flexibility in response to increased cardiac workload has been demonstrated by a decreased PCr-to-ATP ratio in exercising patients with diabetes compared with when at rest (24).

## MODELS OF DIABETIC HEART DISEASE

There are numerous experimental models now available for studying the heart in diabetes, from in vitro systems in isolated cells to in vivo models in small rodents. In addition, larger animal species such as pigs, dogs, and even nonhuman primates have now been characterized for their validity in modeling diabetes. There are important differences between these models in terms of their ability to reproduce key features of diabetic heart disease. Although our primary focus will be on rodent models of diabetes, we will begin by discussing the growing development of more humanized translational cell models using inducible pluripotent stem cell-derived cardiomyocytes.

## IN VITRO MODELS OF INSULIN RESISTANCE AND TYPE 2 DIABETES

### Human Inducible Pluripotent Stem Cell-Derived Cardiomyocytes

Cell models offer valuable tools to study the underlying molecular mechanisms of insulin resistance and T2D in greater detail. In 2006, Takahashi and Yamanaka (29) demonstrated that human somatic cells can be reprogrammed into a developmental “ground state” (i.e., induced pluripotent stem cell, iPSC), before being differentiated and matured into human-iPSC-derived cardiomyocytes (hiPSC-CMs) (30). This exciting breakthrough in cell research has opened new avenues for translational studies, investigating disease mechanisms, high-throughput drug testing, and advancing the potential for personalized medicine.

**Figure 1.**
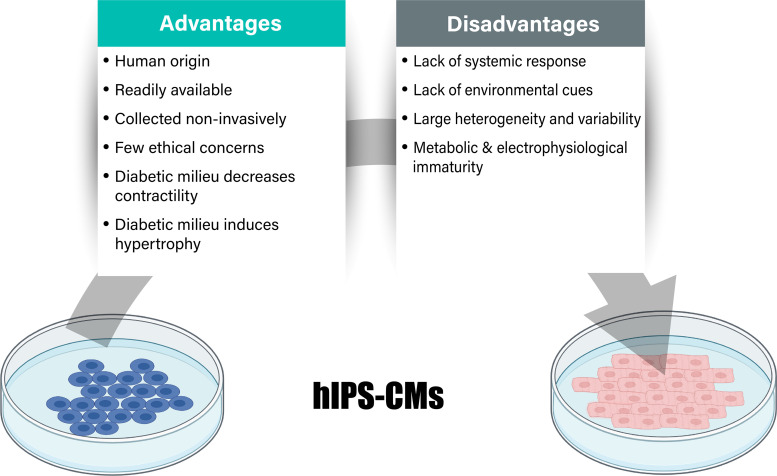
In vitro models of type 2 diabetes (T2D). Advantages and disadvantages of human-induced pluripotent stem cell-derived cardiomyocytes (hiPSC-CMs) with details on key aspects relating to phenotypic features of diabetic heart disease.

The use of hiPSC-CMs to study complex multifactorial, lifestyle-related disorders like diabetes has been minimally explored when compared with monogenic diseases and regenerative medicine. The few available studies primarily make use of healthy donor-derived hiPSC-CMs and expose these to a diabetic-like environment, by mimicking the hyperglycemia, hyperlipidemia, hyperinsulinemia, or other circulating factors including cortisol and endothelin-1 (31–37). An advantage of this approach is that different developmental stages within the diabetes pathogenesis can be mimicked, and the different circulating factors can be independently manipulated (Fig. 1). hiPSC-CMs exposed to high palmitate conditions showed reduced expression of proteins involved in insulin signaling, and reduced insulin-stimulated glucose uptake, together with increased non-insulin stimulated fatty acid uptake (31–33, 37). hiPSC-CMs exposed to a diabetic-like media showed increased hypertrophic markers, cellular hypertrophy, and reduced contractility (35–37). This experimental approach has been used to screen for new therapeutic compounds as well as to investigate the mechanisms of action of existing drugs used in the treatment of diabetes (34–36).

A few studies have generated iPSC-CMs directly from patients with T2D (T2D hiPSC-CMs) (36, 38). One study used skin biopsies from two patients with diabetes, one patient with slow disease progression without cardiovascular disease, and one patient with fast disease progression with cardiovascular disease (36). Both studies showed that iPSC-CMs obtained from patients with diabetes contained features similar to that seen in the diabetic heart, suggesting genomic or epigenomic predisposition to the disorder that is retained during the reprogramming/maturation protocol. T2D hiPSC-CMs exhibited a hypertrophic phenotype, increased brain natriuretic peptide release, and sarcomeric disarray (37, 38). In addition, the patient-derived cells showed increased lipid accumulation and peroxidation, reduced mitochondrial number, abnormal mitochondrial structure, and decreased oxygen consumption rates (38). The cellular changes seem to correspond to the clinical status of the donor (36), although the disease phenotype at the cellular level seems to precede clinical manifestations (38). Therefore, both healthy hiPSC-CMs that are exposed to a diabetogenic environment and iPSC-CMs from patients with T2D show some features like those seen in diabetic cardiomyopathy and therefore may be valuable models to study its pathology.

The advantages of hiPSCs are that they are of human origin, readily available, collected in a noninvasive manner, potentially able to form any cell type, and have relatively few ethical issues. However, there is greater heterogeneity obtained during cell culturing when compared with other widely used in vitro models of insulin resistance and diabetes, such as rodent cardiomyocytes or immortalized cardiac cell lines. Differences in individual donors, genetic stability, and experimental variability (e.g., in reprogramming, differentiation, and maturation protocols) cause variation (39). To account for this variability, the use of multiple patient and control cell lines, as well as multiple clones is recommended. Another limitation of hiPSC-CMs is their relative immaturity on a metabolic, structural, and electrophysiological level when compared with human adult cardiomyocytes. Engineered heart tissue and self-assembling organoids are now being used in the field to provide models to investigate disease pathogenesis as well as for early stage drug testing but are in the infancy of their development and utilization for diabetic heart disease (40–42). Taken together, this hiPSC-CM model approach holds exciting promise for diabetic heart disease research. Technological improvement of hiPSC-CM maturity, standardization of experimental protocols for generating hiPSC-CM models, and creation of three-dimensional engineered heart tissue will help bridge the gap between model and clinical practice. In addition, it is advised to consider the disease development stage of the hiPSC-CM model and to report information on genetic background, possible epigenetic modifiers (e.g., lifestyle), gender, and clinical phenotype of the donor whenever possible.

### Cardiomyocyte Models of Animal Origin

Before the development of iPSCs, most in vitro studies on insulin resistance and diabetes have been performed using neonatal and adult rodent cardiomyocytes. Cells are typically isolated from control animals and exposed to diabetic-like conditions, or cells can be directly isolated from insulin-resistant/diabetic animal models. Alternatively, immortalized rodent cell lines, such as the mouse atrial cell line HL-1 and the rat cardiomyoblast cell line H9C2, have been used following exposure to a diabetogenic milieu.

Immortalized cell lines have unlimited cell renewal capacity and are relatively easy to transfect, enabling molecular biological manipulations. However, they present with a tumor-like metabolic phenotype with suppression of oxidative metabolism and triggered glycolytic metabolism, contrasting that of the adult heart. Approaches to promote an insulin-resistant state in primary and immortalized cardiomyocytes are similar to those used in hiPSC-CMs by exposing them to metabolic stimuli, including high amounts of palmitate (31), glucose (43), insulin (44), and uric acid (45). Furthermore, activators of the inflammatory response like lipopolysaccharide (46) and tumor necrosis factor-α (47) have been used to induce insulin resistance. Paralleling hiPSC-CMs, exposure of rodent cardiomyocytes to diabetic-like conditions generally leads to reduced insulin signaling and insulin-stimulated glucose uptake, a metabolic shift to fatty acid metabolism and lipotoxicity (48). Decreased contractile function has also been demonstrated in adult rat cardiomyocytes exposed to lipid surplus (31). Isolated rodent cardiomyocytes incubated with high concentrations of glucose have demonstrated glucotoxic phenotypes, characterized by increased apoptosis, NADPH oxidase activation, and flux through the polyol and hexosamine biosynthetic pathways (49, 50). Of note, palmitate-induced insulin resistance models of hiPSC-CMs, human embryonic SC-CMs, HL-1 cells, and primary adult rat cardiomyocytes have been compared in the literature (33). Despite their differences in degree of maturation, all model systems showed similar responses to lipid exposure regarding changes in fatty acid uptake, glucose uptake, and insulin signaling.

Primary cardiomyocytes from rodent models of diabetes maintain their diabetic-like phenotype after isolation (51–53), although it is unclear for how long. Although cells that are isolated from a streptozotocin (STZ)-induced rat model of T1D maintain a blunted insulin-stimulated glucose uptake following overnight culturing (43), cells isolated from Zucker obese (ZO) fatty rats appear to lose their metabolic phenotype toward increased fatty acid oxidation after 48 h in culture (54). Caution should therefore be taken when culturing isolated cardiomyocytes of in vivo models of (pre)diabetes, as their diabetic-like phenotype may disappear. Often neonatal cardiomyocytes are used, because of their greater cell yield, ease of transfection, and spontaneous beating when compared with isolation from adult animals (55). However, neonatal cardiomyocytes are more metabolically and functionally immature than their adult cardiomyocyte counterparts.

## IN VIVO MODELS OF INSULIN RESISTANCE AND TYPE 2 DIABETES

### Dietary Manipulation to Induce Insulin Resistance and Type 2 Diabetes

#### High-fat diet-induced obesity (prediabetes).

One of the most frequently used models of obesity/insulin resistance in rodents involves provision of a high-fat diet, with the time frame of dietary provision usually ranging anywhere from as little as 4 wk to as long as 1 year. There are a variety of commercial vendors that supply high-fat diets containing different fat percentages and sources of fat (e.g., saturated fat-based and unsaturated fat-based). One of the most popular vendors in North America is Research Diets, with their 45% kcal from lard and 60% kcal from lard high-fat diets being frequently used to study the pathology of obesity and/or prediabetes.

**Figure 2.**
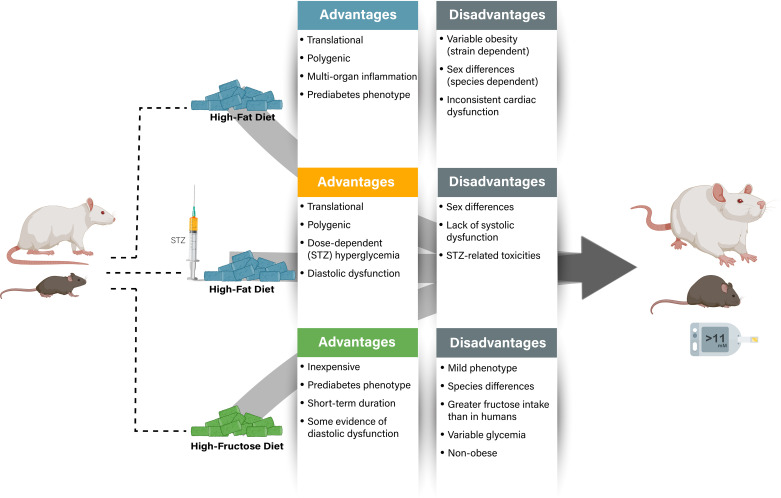
Dietary models of prediabetes/type 2 diabetes (T2D). Advantages and disadvantages of the primary dietary models of prediabetes/T2D, with details on key aspects relating to phenotypic features of diabetic heart disease. While “nonobese” is listed as a disadvantage due to prediabetes/T2D often been associated with underlying obesity, the absence of obesity can also be an advantage if one needs to address whether the cardiac phenotype is independent of body weight gain. STZ, streptozotocin.

The diet-induced obesity (DIO) model is widely used in rodents as it recapitulates numerous features of human obesity, including a progressive weight gain involving expansion of visceral adipose tissue and whole body insulin resistance (Fig. 2) (56). However, there are also a multitude of variables one must consider when using the DIO model. Strain is highly important, as certain strains are resistant to DIO [such as the inbred SWR/J and A/J mice (56, 57)], whereas others are highly susceptible, such as the inbred C57BL/6J strain from Jackson Laboratories, which is one of the most widely used strains for DIO studies (56, 58). Yet despite being highly susceptible to DIO, only a proportion of C57BL/6J mice develop significant obesity in response to high-fat diet supplementation, with males being more susceptible than their female counterparts (59, 60). The most common rat strains used for DIO studies include Sprague–Dawley, Wistar, and Long-Evans rats, but unlike the previously described mouse strains, these rat strains are all outbred, and thus one needs to consider potential genetic variation in the strain between studies. In parallel to their mouse counterparts, Sprague–Dawley rats also demonstrate significant variability regarding the progression of obesity in response to DIO (61, 62), though their progression is much more comparable between males and females (61). Another area of consideration in DIO studies involves the control diet of comparison, as investigators frequently use animal facility standard chow for their lean control mice, which are often included within an institution’s operating animal housing costs. A commercial provider’s DIO-associated control diet will usually be matched to the high-fat diet for micronutrients and protein.

Although obesity is a clear risk factor for the progression of cardiovascular disease in humans, one of the primary concerns with DIO models in animals, particularly in mice, is that they do not consistently exhibit changes in cardiac function in vivo. Many studies have shown no changes in diastolic or systolic function in obese C57BL/6J mice, regardless of the type of diet used to induce obesity (e.g., lard-based, hydrogenated coconut oil) (63–66). Nonetheless, there are inconsistencies in the field with these diets, as others have reported that they do promote cardiac dysfunction (67), and in some cases they induce significant systolic dysfunction (e.g., decreased LVEF) reminiscent of a HFrEF phenotype (68). Similarly, some studies have suggested that high-fat-based DIO models do promote in vivo diastolic dysfunction (69, 70), but this still needs to be evaluated more extensively. It is important to consider how these findings relate to the human population being modeled, and the relationship between cardiac dysfunction and the degree of obesity in patients (71). Numerous mechanisms proposed to contribute to diabetic heart disease are also exhibited in DIO models. This includes alterations in cardiac metabolism [e.g., decreased glucose oxidation and increased fatty acid oxidation (53, 65, 72, 73)], cardiac lipotoxicity [e.g., ceramide and/or diacylglycerol (DAG) accumulation (63, 67, 74)], inflammation (75), microvascular dysfunction (76), and endoplasmic reticulum stress (77, 78) to name a few. The fact that these mechanisms are also evident in rodent models of T2D would suggest that they are induced by obesity and may be directly causal to the pathology of diabetic heart disease.

#### High fructose-induced insulin resistance/prediabetes.

A high-fructose dietary intervention has proven to be a useful experimental tool to generate a preclinical rodent model to study cardiac pathology associated with insulin resistance and prediabetes (Fig. 2). These dietary studies have demonstrated that dietary fructose confers adverse effects on systemic metabolism and cardiac function, even in the absence of marked hyperglycemia and obesity (for review, see Ref. 79). Tightly controlled dietary intervention studies comparing fructose, glucose, and sucrose have advanced the proposition that it is the fructose component of the sucrose dimer that mediates the adverse metabolic response to sugar-induced metabolic disease (80). The characteristics of fructose-induced systemic metabolic dysregulation in rodents may be species-specific, and caution must be taken when comparing between species, but in most settings rats are more susceptible than mice. In general, the systemic phenotype induced by a high-fructose diet in rodents most commonly includes insulin resistance and dyslipidemia (81–83), with fructose-induced hypertension and hyperinsulinemia also reported in some studies (84, 85). Blood glucose is either unchanged or mildly elevated in fructose-fed rats and mice, recapitulating the very early stages of disease progression (86, 87).

Reported methodology for fructose dietary interventions varies in diet composition (% fructose, iso- vs. hyper-caloric), administration (pellets vs. drinking water), and diet duration. Typically, studies in rats and mice use isocaloric diets containing 60%–72% of energy from fructose, for 4–12 wk duration. A custom-control diet matched to the fructose diet for energy, macronutrients and micronutrients allows for a tightly controlled dietary intervention where the effects of dietary fructose can be directly investigated. Administration of the diet using pelleted food generally provides stable food intake and ensures an isocaloric setting. Interventions that add fructose to the drinking water (usually 10% fructose) may result in higher (or variable) caloric intake, depending on whether extra calories ingested via the drinking water are offset by a reduction in food intake. Housing conditions can also have an effect, and an important consideration for fructose dietary studies is the humidity of the animal housing unit. High humidity can affect the consistency of the fructose diet pellets, and frequent replacement of food is required to maintain palatability (e.g., every 1 to 2 days). To preserve the integrity of the fructose diet, the pellets should be refrigerated (or frozen for long-term storage) in sealed containers because of the susceptibility of high sugar content to support bacterial growth.

The key practical advantages of the fructose diet model of prediabetes are that it is relatively inexpensive, short term, and can be easily applied to rodent models with underlying genetic conditions. The model recapitulates clinical features of insulin resistance and allows investigation into the impact of a relatively mild prediabetic state, in the absence of marked hyperglycemia and obesity. Despite this mild systemic phenotype, a notable cardiac phenotype has been reported. Cardiac tissue insulin resistance is evident as demonstrated by downregulation of the insulin signaling pathway in fructose-fed rodents (86). A high-fructose diet has also been shown to induce cardiac hypertrophy, oxidative stress, and apoptosis (82, 88, 89). Fructose-induced cardiac dysfunction has been characterized by alterations in cardiomyocyte excitation-contraction coupling and Ca^2+^ handling (90). Although not yet extensively studied, some evidence of diastolic dysfunction in fructose-fed rodents has been reported using flow-Doppler echocardiography (*E/A* ratio) (91, 92). However, further work is required to fully characterize the structural and functional changes that occur in response to a high-fructose diet using in vivo imaging modalities.

### Combination Dietary Manipulation and Pharmacology to Induce Insulin Resistance and Type 2 Diabetes

#### High-fat diet/streptozotocin model of type 2 diabetes.

Although numerous dietary strategies are used to model insulin resistance and/or a prediabetic state in rodents, the addition of low-dose STZ injections (usually around 25 mg/kg in rats and 75 mg/kg in mice) to a modified diet is becoming increasingly popular. STZ is a β-cell toxin, typically used at higher concentrations to produce T1D (see *Streptozotocin Model of Type 1 Diabetes*); however, when used at a lower dose in combination with a high-fat diet, it can induce hyperinsulinemia and hyperglycemia. This model imparts several practical advantages including its lower cost, its speed to develop the disease, and the ability to modify the STZ dose to generate a spectrum of disease severity (Fig. 2). The diet incorporated is often a high fat or a combination of high fat and high sucrose provided anywhere from 3 to 12 wk in duration, with STZ administered at ∼2 wk in rats, and at ∼4 or 5 wk in mice. Solutions of STZ should be prepared freshly in a citrate buffer at pH 4 and used immediately, because STZ rapidly precipitates out of solution (93). Fasting animals (for at least 5 h or overnight) before STZ administration increases reproducibility, by minimizing competition between STZ and blood glucose for the islet β-cell glucose transporter (GLUT) 2 (56). The amount of STZ can be titrated to induce the extent of hyperglycemia required and can either be given as a one-off bolus or on multiple occasions. For example, in Wistar rats, an STZ concentration below 30 mg/kg induces modest increases in plasma glucose while maintaining the hyperinsulinemia produced by the high-fat diet, but above 30 mg/kg the hyperglycemia becomes more severe and is accompanied by hypoinsulinemia, weight loss, and hyperketonemia (94). When one uses this model for the first time, it is recommended that a pilot study is carried out to optimize the dose of STZ needed, as strain, sex, and housing environment can influence susceptibility.

The addition of low-dose STZ to a high-fat diet elicits a highly reproducible diastolic dysfunction (4, 95–98), and manifests as a reduction in the mitral *E*/*A* and tissue Doppler *e′*/*a′* ratios, or an elevation in the *E*/*e′* ratio, the latter of which is often more reliable and reproducible than the former (99). Using the isolated perfused heart, diastolic dysfunction is evident in response to stress such as hypoxia, which induces an increase in end-diastolic pressure and can be reversed by treatment with metabolic therapy (100). Of interest, the diastolic dysfunction observed in males does not appear to be evident in females, though this could be due to female C57BL/6J demonstrating resistance to the actions of STZ (101, 102), and this will need to be further characterized in future studies.

Despite the dietary/low-dose STZ model producing a highly reproducible diastolic dysfunction in rodents, for the most part, LV systolic function remains normal when compared with lean, healthy controls (96, 97), but whether prolonged duration of the model yields systolic dysfunction remains to be determined. In unpublished findings by the Ussher group, male and female C57BL/6J mice following 18 wk of dietary supplementation with a high-fat diet (STZ administered at the 5-wk time point) are still devoid of any notable systolic dysfunction. Although baseline cardiac function is normal in rodents subjected to the dietary/low-dose STZ model, the response following ex vivo ischemia-reperfusion is abnormal, with decreased recovery of rate pressure product (34). However, it has not been determined how rodents subjected to this model recover following in vivo myocardial infarction or heart failure produced via surgical intervention.

Importantly, the dietary/low-dose STZ model in rodents recapitulates a number of molecular mechanisms indicative of diabetic cardiomyopathy in humans, including impaired energetics and mitochondrial dysfunction (103, 104), often accompanied by elevations and reductions in myocardial fatty acid oxidation and glucose oxidation, respectively (94–97, 100). In addition, this model presents with impaired metabolic flexibility in response to stress, including an impaired ability to upregulate glycolytic flux in response to both acute and chronic oxygen restriction (100, 105). Increases in cardiac fibrosis, inflammation, and oxidative stress, as well as impaired calcium handling are also observed in hearts of rodents subjected to dietary/low-dose STZ-induced T2D (103, 106, 107). This model is highly responsive to most major therapies used in the treatment of T2D, including the first-line therapy metformin (108), the glucagon-like peptide-1 receptor (GLP-1R) agonist liraglutide (95), and the sodium-glucose cotransporter-2 (SGLT2) inhibitor empagliflozin (109), verifying its validity as a translational model. However, a limitation with this model is the inherent toxicities associated with STZ use (e.g., hepatic genotoxicities). There is also a dearth of published information with this model in female rodents, as well as in older rodents, and it will be important for investigators to take this into consideration, given the demographic of individuals affected by T2D.

### Genetic Models of Insulin Resistance and Type 2 Diabetes

#### ob/ob and db/db mouse models of type 2 diabetes.

**Figure 3.**
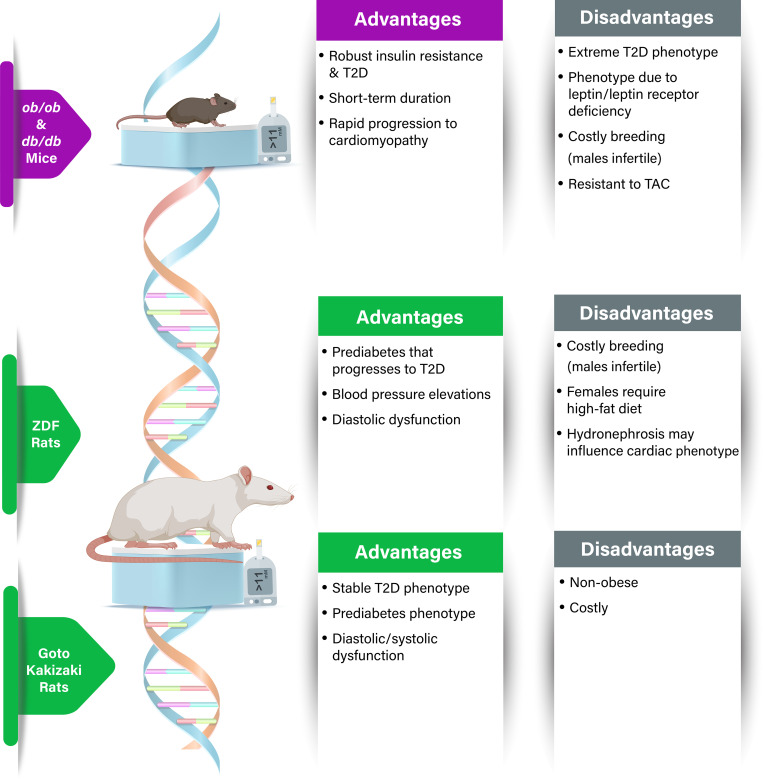
Genetic models of type 2 diabetes (T2D). Advantages and disadvantages of the primary genetic models of T2D, with details on key aspects relating to phenotypic features of diabetic heart disease. While “nonobese” is listed as a disadvantage due to T2D often been associated with underlying obesity, the absence of obesity can also be an advantage if one needs to address whether the cardiac phenotype is independent of body weight gain. TAC, transverse aortic constriction.

There are two genetic mouse models of T2D that are frequently used to study the pathophysiology of diabetic heart disease: the obese *ob/ob* mouse and the diabetic *db/db* mouse (Fig. 3) (110). Leptin is a peptide hormone secreted by mature adipocytes that regulates food intake and energy expenditure, and these two mouse models are congenitally deficient in either leptin (*ob/ob*) or the leptin receptor (*db/db*). Consequently, both the *ob/ob* and the *db/db* mice are hyperphagic with development of severe obesity due to increased energy intake and reduced metabolic rate. The increase in body weight plateaus at ∼2 mo of age, approximately double the weight of their lean genetic controls. Although there may be some quantitative and chronological differences, the overall metabolic alterations appear very similar in both *ob/ob* and *db/db* mouse strains (111), but with the *db/db* being more extreme phenotypically. Both models exhibit early signs of hyperinsulinemia due to insulin resistance, euglycemia which progresses to overt hyperglycemia, and dyslipidemia which presents by ∼15 wk of age (111).

These two models exhibit a cardiac metabolic phenotype with high reliance on myocardial fatty acid oxidation, reduced glucose oxidation rates, mitochondrial dysfunction, and impaired insulin-stimulated glucose uptake (112–114). Another consistent finding is impaired cardiac energetics with reduced energy status (115, 116). Studies addressing the progression from prediabetic state (around 4–6 wk of age) to overt insulin-resistance state (around 12–15 wk of age), suggest that the alterations in myocardial metabolism precede the development of ventricular dysfunction (111, 117, 118), findings which are concordant with observations made in clinical studies (20). Studies on cardiovascular pathophysiological processes in older *db/db* and *ob/ob* animals report common traits of diabetes-induced cardiovascular pathology such as increased vascular and myocardial oxidative stress, fibrosis, apoptosis, impaired calcium handling, endothelial dysfunction, and impaired vascular compliance (112, 119–124). As these models are quite severe, the progression to cardiac complications is rapid in these genetic models, facilitating time-effective studies when compared with less severe models of obesity-induced insulin resistance. Careful assessment of ex vivo ventricular function in older *db/db* mice with established hyperglycemia reports LV systolic and diastolic dysfunction with reduced cardiac output and impaired parameters of ventricular function (116, 125, 126). In vivo assessment of cardiac function with echocardiography or MRI are not conclusive, reporting both impaired (118) and unaltered in vivo cardiac function (115, 126) in *db/db* mice, which could be explained by systemic adaptations to sustain cardiac function.

The *db/db* mouse has been used extensively to study antidiabetic treatments, including established drugs used in clinical practice (e.g., biguanides, α-glucosidase inhibitors, sulfonylureas, thiazolidinediones, dipeptidyl peptidase-4 inhibitors, GLP-1R agonists, and SGLT2 inhibitors), as well as experimental compounds. Branded drugs are commonly used as positive controls and produce plasma glucose and insulin lowering effects in *db/db* mice (127–129). Of interest, the effects on hyperglycemia of these antidiabetic drugs are not always sufficient to preserve insulin secretory capacity in the *db/db* mouse (129), most likely due to the severity of this model. In terms of cardiovascular effects, many studies have shown that antidiabetic treatments attenuate cardiac pathological remodeling in *db/db* mice (128, 130–133), even in the absence of glucose-lowering effects (134).

The *db/db* and the *ob/ob* models are extreme models of hyperphagia, obesity, and eventually uncontrolled diabetes, and the progression from obesity to β-cell dysfunction in the *db/db* mouse is extremely rapid when compared with patients (and mechanistically most patients with obesity/diabetes do not have genetic mutations in leptin or its receptor). If planning longer-term treatment/aging studies in the *db/db* mouse, care must be taken in older mice as they develop additional systemic complications, which challenges both housing and handling of these animals. They show early signs of cold intolerance, are susceptible to several stressors, and with age they frequently develop subcutaneous inflammation, abnormal liver, kidney and spleen morphology, weight loss, lymphomas, peripheral neuropathy, and poor wound healing. This can have practical implications, for example, limiting tail vein cannulations in older mice to avoid tail necrosis and consideration of the humane endpoint for terminating experiments. There are also challenges concerning breeding as these monogenic obese animals are functionally infertile, which makes breeding between the heterozygotes costly.

#### Zucker diabetic fatty rat model of type 2 diabetes.

This obese T2D model originates from the identification by Theodore and Lois Zucker of a missense mutation in the leptin receptor gene in outbred Merck-M rats, leading to the development of obesity. This strain was named Zucker-Lepr*^fa/fa^* and is commonly referred to as the fatty or ZO rat. Selective inbreeding of ZO rats over several generations led to the identification of a new substrain of ZO rats with a more diabetic-like phenotype referred to as the Zucker diabetic fatty (ZDF) rat. When compared with their lean equivalents, namely, lean fa/+ or lean Zucker (LZ) rats, ZO rats exhibit increased caloric intake from the age of 4 wk and progressively develop obesity (135). From 6 to 7 wk of age, sustained hyperinsulinemia is sufficient to maintain normoglycemia in most ZO rats, with some reports demonstrating a mild to moderate increase in blood glucose in some ZO rats later in age (136, 137). In contrast, ZDF rats become insulin resistant at 6 to 7 wk of age and have elevated concentrations of insulin (138, 139), which progresses to a robust hyperglycemia by 10–14 wk of age (138, 140). As ZDF rats age, an imbalance between β-cell hyperplasia and apoptosis results in the progressive decrease of insulin secretion, paralleled by an increase in glycemia that can reach >700 mg/dL at 24 wk of age (141, 142). Accordingly, ZDF rats reproduce the course of human T2D from the prediabetic to the diabetic state, as well as other key features of the human metabolic syndrome including dyslipidemia (142, 143) and a mild elevation in blood pressure (Fig. 3) (144).

Unfortunately, there has been a relative lack of comparison between male and female ZDF rats, likely because the spontaneous diabetic phenotype in males is not observed in female ZDF rats. Despite female ZDF rats being obese and hyperinsulinemic, they need to be fed an obesogenic diet to induce T2D (141, 145). The recommended obesogenic diet comprises 48% kcal from fat, which if provided to females induces T2D and if provided to male ZDF rats will further exacerbate their diabetic phenotype (145). The mechanisms explaining these sex-specific differences in diabetes susceptibility remain unknown and will need to be resolved. This is particularly important if using ZDF rats as a model for studying diabetic cardiomyopathy, as in humans diabetic cardiomyopathy and HFpEF are more prominent in women with T2D than in men (146). From 2 mo of age, male ZDF rats demonstrate decreased heart capillary density, and an increase in cardiomyocyte area by 8 mo of age (141, 144). In contrast, echocardiography analysis demonstrates that ZDF rats exhibit no changes in LV end-diastolic diameter or septal and posterior wall thickness (143), and no changes in heart weight-to-tibia length or body weight ratios have been reported in multiple studies using male ZDF rats (138, 143, 147). Millar catheter pressure/volume studies identified the presence of diastolic dysfunction in ZDF male rats at 16 wk of age, which was further exacerbated at 36 wk of age and accompanied by an increased end-diastolic volume (147). Similarly, diastolic dysfunction has also been reported in ZDF rats at 44/45 wk of age, reflected by an increase in LV end-diastolic pressure (148). Of interest, diastolic dysfunction and cardiac hypertrophy have been identified in female ZDF rats, but only when fed on an obesogenic diet (141).

ZDF male rats also recapitulate the changes in myocardial metabolism characteristic of diabetic cardiomyopathy, including decreased rates of glucose metabolism (149, 150), as well as an increase in palmitate metabolism (151). The increased palmitate oxidation in ZDF rats is unable to match the elevated rate of palmitate uptake, such that myocardial triacylglycerol (TAG) and other fatty acid intermediates such as DAG and ceramide accumulate, mediating cardiac lipotoxicity. Of clinical relevance, ZDF rats do respond to the majority of antidiabetic therapies including metformin (152), the GLP-1R agonist liraglutide (153), and the SGLT2 inhibitor empagliflozin (154), highlighting their utility as a translational model of T2D.

An important limitation in using ZDF rats relates to their cardiac phenotype being potentially dependent on their spontaneous development of hydronephrosis, which is characterized by a swelling of the kidney due to an obstructed bladder, a clinical symptom that is absent in humans with T2D (147, 155). Moreover, LV systolic wall stress positively correlates with blood urea nitrogen levels, consistent with the notion that the diabetes-related cardiac dysfunction in ZDF rats may be biased by the presence of underlying kidney disease (155). Therefore, it is recommended that users of the ZDF rat strain rigorously evaluate renal morphology and function to exclude the presence of hydronephrosis as a confounding factor when evaluating the onset of diabetic cardiomyopathy. Similar to what has been previously described for *db*/*db* mice, ZDF rats are functionally infertile, which makes breeding costly as heterozygotes (Zucker-Lepr*^fa/+^*) are needed to maintain the strain.

#### Goto–Kakizaki rat model of type 2 diabetes.

The Goto–Kakizaki (GK) rat was generated by selective breeding of Wistar rats over numerous generations with glucose intolerance used as a selection index (156). The GK rats manifest the major features of the metabolic, hormonal, and microvascular disorders described in T2D (Fig. 3). GK rats show basal hyperglycemia, hyperinsulinemia, and impaired secretory response to glucose as early as 3 to 4 wk of age, and insulin resistance, dyslipidemia, and T2D at ∼12–16 wk of age (157–159). There are no apparent differences linked to sex regarding basal circulating glucose and insulin levels in GK rats (159), and nondiabetic Wistar rats are the optimal control group for comparison. The GK rats show β-cell dysfunction even in the absence of glucose intolerance from the first 3 wk after birth, a prediabetes period, which cannot be reversed by normal postnatal nutrition and nursing behavior, indicating a genetic basis of T2D (159, 160). They also display late diabetic complications, such as neuropathy (161), retinopathy (162), and polycystic ovary syndrome (163). Genetic linkage analysis suggests that distinct combinations of genetic loci are responsible for different physiological characteristics associated with the diabetic phenotypes, which is consistent with the features of polygenic T2D in humans (164, 165).

Cardiac morphology and function of the GK rat largely mimic those of patients with T2D, including hypertrophy, contractile dysfunction, and metabolic alterations. Cardiac hypertrophy assessed by heart weight to body weight ratio, wall thickness by echocardiography, and individual cardiomyocyte size by histology can be detected as early as at 8 wk of age (166). Functional contractile analyses have generated mixed results. Female GK rats have reduced myocardial blood flow and contractility at the age of 8 to 13 mo, as assessed by perfusion and cine MRI (167). Ultrasound echocardiography studies have demonstrated that male GK rats have preserved systolic but reduced diastolic function at 6 to 7 mo of age, indicative of a diabetic cardiomyopathy phenotype (168, 169), whereas systolic function declines at 12 mo of age (170). Other studies show preserved basal contractility in isolated perfused hearts (171) and in isolated ventricular myocytes (172). Despite this, male GK rats show increased susceptibility to ischemic injury in isolated perfused hearts (171, 173) and accelerated cardiac remodeling after myocardial infarction (174). The male GK rat shows decreased myocardial glucose utilization and nearly a twofold increase in fatty acid oxidation (169). A limitation of the GK rat is that they are costly and nonobese, with minimal fat accumulation in the liver (160, 175). This absence of obesity does not replicate the classical presentation in most patients. However, feeding the GK rat with a high-fat diet can be an alternative approach to induce obesity, which exacerbates the defects in metabolism and cardiac ultrastructure (176, 177).

#### Other monogenic models of type 2 diabetes.

Several other mouse models of T2D have been used over the years with various benefits and limitations, some of which have value in replicating aspects of diabetes but have not yet been examined extensively for cardiovascular phenotypes. The KKA*^y^* mouse was first described in 1970 (178) and generated from the spontaneously diabetic Kuo Konodo (KK) mouse (179), which was bred to also carry the yellow obese gene (A*^y^*) (178). The KKA*^y^* mouse model has predominantly been used to study diabetic kidney disease (180, 181), with only a few studies fully exploring its impact on diabetic cardiomyopathy (182). This model has been shown to respond to empagliflozin treatment, thereby improving cardiac fibrosis and oxidative stress markers (182).

More recently, the TallyHo (TH) mouse strain was derived from mice showing polyuria and glucosuria, with genetic mapping identifying three quantitative trait loci focused on regions of chromosome 19 including *Tanidd1*, chromosome 18 *Tanidd2*, and a locus on chromosome 16 (183). Relatively little work has been carried out on the cardiovascular phenotype of these mice, making them another novel model for potentially studying the impact of T2D on the heart. A limitation of the TH mouse is its relatively late onset of hyperglycemia [∼26 wk of age (183)] and that only males develop overt hyperglycemia, which is not 100% penetrant (184).

Other investigators have focused on specific aspects of diabetes and have generated targeted approaches to specifically define metabolism and signaling in the heart, such as overexpression or loss of function of key metabolic regulators (e.g., peroxisome proliferator-activated receptor α, insulin receptor) and have been previously reviewed in detail (185, 186). Finally, in the attempt to address precision medicine approaches, investigators have examined common monogenic mutations that are linked to obesity and diabetes. One example is the generation of mouse models for mutations in the melanocortin 4 receptor, a G protein-coupled receptor. Specifically, the recent development of melanocortin 4 receptor hypermorphic mice revealed obesogenic and diabetogenic effects but did not characterize heart function (187).

## IN VIVO MODELS OF TYPE 1 DIABETES

### Streptozotocin Model of Type 1 Diabetes

STZ is derived from the Gram-positive bacterium *Streptomyces achromogenes*, and primarily damages insulin-producing pancreatic β-cells because of its affinity for GLUT2, producing a T1D phenotype (188, 189). As described in combination dietary manipulation and pharmacology to induce insulin resistance and type 2 diabetes, STZ must be freshly prepared in acidic citrate buffer and administered in the fasted state to increase the homogeneity of diabetes. The detailed methodology for STZ treatment for mice and rats has been elaborated in a recent publication (190). It is important to note that mice and rats have different susceptibilities to STZ-induced diabetes, with mice requiring a higher dose than rats. There are two frequently used methods of STZ administration to induce T1D, which include either a single, high-dose treatment or a multiple, lower-dose treatment protocol. In mice, a single intraperitoneal injection of 200 mg/kg STZ can induce hyperglycemia, whereas, in rats, a single dose of 65 mg/kg STZ can be sufficient (190). This method increases blood glucose levels to >500 mg/dL within 2 days through nearly complete destruction of pancreatic β-cells (191). As high-dose STZ causes massive and rapid β-cell death, the risk of mortality within the initial 24 h postadministration is high, therefore, animals must be monitored closely. In addition, 10% sucrose water should be provided following STZ administration, to help avoid hypoglycemia caused by the destruction of pancreatic β-cells. To slowly develop a T1D phenotype, which more closely mimics an autoimmune insulitis, low-dose (40–50 mg/kg body wt in mice) intraperitoneal injections of STZ for five consecutive days can be used (190). This method causes less STZ-induced toxicity and has reduced mortality risk (and supplementation of sucrose water can also be applied to this approach).

**Figure 4.**
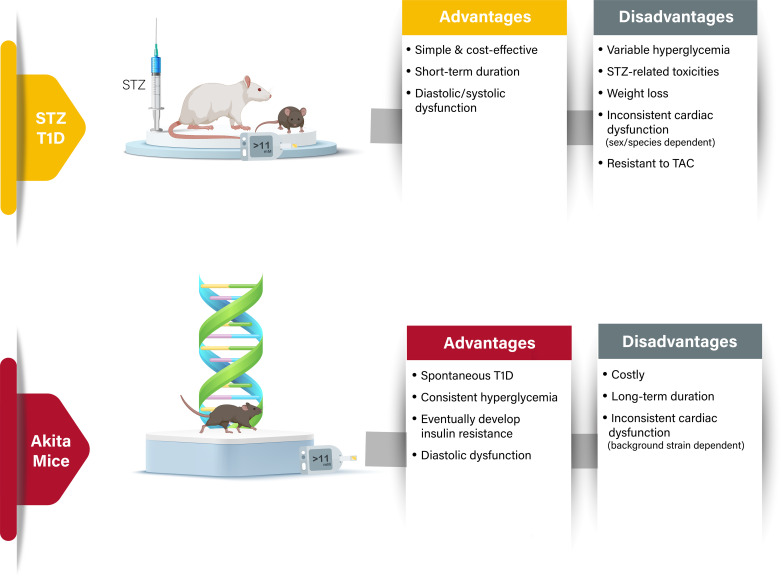
Models of type 1 diabetes (T1D). Advantages and disadvantages of the primary models of T1D, with details on key aspects relating to phenotypic features of diabetic heart disease. T2D, type 2 diabetes; TAC, transverse aortic constriction.

One of the major advantages of the STZ model of T1D is that it is simple, cost-effective, and expeditious (Fig. 4) (192). However, one must consider when using this model that the hyperglycemic effect of STZ can be highly variable within a group of animals, and that STZ sensitivity varies between different strains, where some are high responders and others are low responders (193). The weight loss induced by high-dose STZ can be extreme, so it may be worth using animals with a higher starting body weight, and carefully considering the window of time between STZ administration and final experiments. Although STZ predominantly affects islets of the pancreatic core, its cytotoxic effects are not restricted to pancreatic β-cells but can also affect the liver and kidney (194–196). In addition, STZ has T1D-independent cardiotoxic effects that need to be considered if using this model to study T1D-related cardiomyopathy (197). STZ can have genotoxic effects such as DNA methylation, DNA strand breaks, and inhibition of DNA synthesis (188). Another important aspect of consideration involves the timing of STZ injection, which can affect the progression of hyperglycemia in animals (strongest at 4:00 PM and weakest at 8:00 AM) (198). There also appears to be sex-specific differences regarding susceptibility to STZ, as female mice are less sensitive to STZ-induced β-cell toxicity (199–201).

Regarding the relevance of STZ-induced T1D in modeling diabetic heart disease, it produces a cardiomyopathy with increased nuclear chromatin condensation and mitochondrial swelling in cardiomyocytes, as well as a marked increase in lipid droplets (202, 203). The metabolic profile of the heart in T1D (increased fatty acid oxidation and decreased glucose oxidation) is also evident in both mice and rats subjected to STZ-induced T1D (116, 204–207). Although female mice may be less sensitive to STZ-induced β-cell toxicity and associated hyperglycemia, the progression of both systolic dysfunction (decreased LVEF) and diastolic dysfunction (increased *E*/*e′* ratio and decreased *e′*/*a′* ratio) appears to be more noticeable than in their male counterparts (208). In general, the overall decrease in LVEF and ensuing systolic dysfunction observed in STZ-treated mice is relatively mild (204, 208), though another study using male Wistar rats was reminiscent of an HFrEF phenotype (209). STZ-induced T1D in male Sprague–Dawley rats also results in systolic dysfunction assessed using pressure-volume loop analysis with Millar catheters, as reflected by decreases in LV pressure and the maximal rate of LV pressure rise (210). Conversely, STZ-induced T1D in male Wistar rats did not produce any systolic dysfunction (decreased LVEF) or diastolic dysfunction (increased *E*/*e*′ ratio and decreased *e′*/*a′* ratio) as assessed by ultrasound echocardiography (211). Similarly, ultrasound echocardiography analysis did not reveal any notable systolic dysfunction (decreased LVEF) in male and female mice subjected to STZ-induced T1D (212). Despite some of these inconsistencies in cardiac function profiles, the STZ model of T1D has been invaluable for investigations of T1D-induced structural, metabolic, and functional remodeling in the heart.

### Akita Mouse Model of Type 1 Diabetes

Insulin 2 heterozygous (*Ins2^+/−^*) Akita mice are a spontaneous, genetic, and nonobese model of T1D, due to a mutation in *Ins2* gene that causes a disruption in normal folding of proinsulin, thereby inducing endoplasmic reticulum stress and subsequent β-cell toxicity and loss. As such, Akita mice exhibit decreased pancreatic β-cell density and elevated blood glucose levels starting from the age of 3 to 4 wk, achieving robust (>500 mg/dL) hyperglycemia at the age of 8 wk (213). Females show milder symptoms compared with males with a marked increase in longevity (690 days of age in females vs. 305 days of age in males) (213). Akita mice are commercially available from the Jackson Laboratory, and careful monitoring of their blood glucose levels is required. Blood glucose levels often fluctuate in female Akita mice whereby transient hyperglycemia is often seen during puberty, which undergoes remission to more moderate hyperglycemia after sexual maturation (213).

Akita mice offer several advantages for studying T1D, with one of the most notable being that they require no form of pharmacological or surgical intervention to induce a T1D phenotype (Fig. 4). Moreover, homogeneity of blood glucose concentrations in Akita mice is notable, with their blood glucose levels being very consistent among different age groups, particularly in males. Akita mice also demonstrate significant relevance to human T1D, as their diabetic phenotype progresses with age in a manner that closely recapitulates the pathogenesis in humans. Therefore, Akita mice are of use to study the pathogenesis and progression of T1D in a chronic fashion in different organs (214), such as the heart (215–218), kidney (219), and liver (220, 221). They are frequently used as a model to study the effect of insulin or antidiabetic drug treatments (219, 222–225). Another advantage of the Akita mouse is that insulin resistance begins to manifest by 13 wk of age (226), suggesting that Akita mice exhibit phenotypes observed in patients with advanced-stage T1D and insulin deficiency combined with insulin resistance. When using Akita mice, it is important to take into consideration the strain. Akita mice are available in two strains, the *Ins2^+/−^* Akita mice on a C57BL/6J background, and the *Ins2^+/C96Y^* Akita mice with a C96Y mutation in the *Ins2* gene on a 129/SvEv and DBA/2 background, the latter of which demonstrates enhanced kidney injury (227). As kidney function can influence heart function, it is important to put in context the results and mention the Akita strain used in experiments.

Importantly, Akita mice exhibit several features that are observed in diabetic cardiomyopathy in humans. This includes increased lipotoxicity, metabolic remodeling, and mitochondrial dysfunction leading to cardiac hypertrophy, fibrosis, and cardiac dysfunction (215, 216, 228, 229). In terms of cardiac function, 20-wk-old male Akita mice (background strain not specified) demonstrate normal systolic function (LV fractional shortening) as assessed via ultrasound echocardiography (230). Certain parameters of systolic function continue to remain normal (LVEF and stroke volume) even at 1 year of age as assessed via invasive pressure-volume conductance catheters, though reductions in cardiac output were observed (230). Similarly, 4-mo-old Akita mice (129/SvEv and DBA/2 background) also exhibit normal systolic function (LVEF and fractional shortening) (231). Conversely, 4-mo-old male Akita mice (C57BL/6J background) have decreased systolic function (LVEF) (232). These inconsistencies in cardiac function profiles once more emphasize the importance of specifying background strain when using Akita mice. Diastolic dysfunction also appears to be present in Akita mice regardless of background, as tissue Doppler assessments indicated reductions in the *e*′/*a*′ ratio at 16 wk of age in Akita mice on a C57BL/6J background (229), while the *E*/*e*′ and deceleration time were increased in 3-mo-old Akita mice on a 129/SvEv and DBA/2 background (215).

## DIABETES-RELATED ATHEROSCLEROSIS FOR STUDYING DIABETIC HEART DISEASE

### High-Fat/High-Cholesterol Diet in Apolipoprotein E or Low-Density Lipoprotein Receptor Knockout Mice

Although the previous sections provided key details on models of insulin resistance and diabetes that can lead to various forms of diabetic cardiomyopathy, none of these models in isolation produce an atherosclerotic-mediated cardiovascular disease. This is clinically relevant since an increased burden of atherosclerosis is recognized as an essential contributor to increased risk of cardiovascular disease in diabetes (8). Wild-type mice predominantly carry cholesterol in high-density lipoprotein (HDL) fractions and are thus protected from spontaneous atherogenesis. Genetic engineering targeted to disrupt clearance of cholesterol-rich lipoprotein particles together with a Western-style, high-cholesterol diet is needed to increase the exposure of vessels to lipid-rich particles, thereby allowing atherosclerotic lesions to reproducibly develop in the aortic sinus and throughout the aortic arch, proximal aorta, and trunk of the brachiocephalic artery (extensively reviewed in Refs. 233 and 234). Elimination of the low-density lipoprotein (LDL) receptor in mice (*Ldlr^−/−^* mice), which increases cholesterol within the LDL particles, or genetic deletion of apolipoprotein E (*Apoe^−/−^* mice), which prevents the clearance of TAG-rich postprandial lipoproteins, have both proven to be invaluable tools in the pursuit of molecular mechanisms influencing atherogenesis (235–237). Systematic evaluation of risk factors for atherosclerosis and validation of several human genome-wide association candidates involved in coronary artery disease have determined that these mice recapitulate most human-related mechanisms for atherosclerosis (238).

To study diabetes-related atherosclerosis, both *Ldlr^−/−^* and *Apoe^−/−^* mice are frequently subjected to the dietary and/or pharmacological models of diabetes described in previous sections. Although the exact composition of “Western style,” atherogenic diets often varies, atherosclerotic lesion development in the aortic arch and throughout the aorta did not differ between high-fat versus high-fat, high-sucrose dietary supplementation (239). However, the addition of sucrose increases insulin resistance, inflammation in peripheral tissues, and accelerates lesion onset (239). *Ldlr^−/−^* mice gain weight, develop adiposity, dysglycemia, and hyperinsulinemia more significantly than *Apoe^−/−^* mice when fed a high-fat, high-carbohydrate diet (240). The breeding of *Apoe^−/−^* mice with T1D Akita mice induces dysglycemia, decreases body weight (both lean and fat mass), but increases fasting and fed cholesterol levels while producing a more severe atherosclerosis (221). Similarly, male *Ldlr^−/−^* mice bred with Akita mice have elevated cholesterol and atherosclerosis relative to *Ldlr^−/−^* mice (241). Hyperglycemia can also be induced by treating *Ldlr^−/−^* mice with STZ (50 mg/kg body wt via intraperitoneal injection for 5 days), which also heightens hypercholesterolemia without significant changes to circulating TAG levels (242). Diabetes induced in *Ldlr^−/−^* mice with viral-mediated destruction of insulin-producing cells also exhibit hypercholesterolemia, accelerated lesion initiation, and advanced stages characterized by intraplaque hemorrhage (243). Studies with hyperglycemia in the absence of differences in plasma cholesterol have identified that diabetes can change the morphology of the plaque with more significant calcification in the proximal aorta (244). Glucose-oxidized LDL also influences monocyte proliferation and migration, suggesting a more complex intertwining between these risk factors (245). It is worth remembering though that atherosclerotic lesions in humans are prothrombotic and result in myocardial ischemia. However, this does not occur spontaneously in the plaques of *Ldlr^−/−^* or *Apoe^−/−^* mice without other manipulations (further reviewed in Ref. 246), such as cross-breeding *Apoe^−/−^* mice with scavenger receptor class B type 1 knockout mice, which results in spontaneous myocardial infarction (247). In addition, despite plaque development, neither *Ldlr^−/−^* nor *Apoe^−/−^* mice develop severe cardiac dysfunction nor do they exhibit abnormal hemodynamic parameters (248). Cardiac hypertrophy has been reported in some studies in aged *Apoe^−/−^* mice, which is worsened if the animals are fed a high-fat diet (249).

## EVALUATION OF CURRENT LITERATURE

We have also performed an extensive evaluation of previous research, published by the *American Journal of Physiology-Heart and Circulatory Physiology*, relating to the use of the aforementioned rodent models of diabetes and insulin resistance. Our initial search [performed by J. R. Ussher (April 23, 2022), followed by secondary searches by L. C. Heather and E. E. Mulvihill for confirmation (April 26, 2022)] focused on articles published from 2020 to 2022 using “diabetes” as our key word on the journal website. This search yielded 256 articles, many of which were excluded for not being “original research articles,” being performed in larger animal models, humans, or other models not characterized in this review, or published before 2020. Following the application of these criteria, our search identified 24 original research articles to include in our evaluation (Table 1) (64, 106, 209, 212, 250–269).

**Table 1.** Evaluation of publications on diabetes published by the American Journal of Physiology-Heart and Circulatory Physiology

Criteria	Results and Comments
Model of diabetes	Total: 24 publications: high-fat diet alone in mice/rats (12 publications), high-fat diet plus low-dose STZ in mice/rats (2 publications), high-fructose diet in mice/rats (1 publication), *db*/*db* mice (2 publications), ZDF rat (1 publication), *Apoe^−/−^* mice plus high-fat diet (2 publications), and STZ in mice/rats (4 publications).
Rationale provided for selection of diabetes model	Vast majority of studies provided a rationale centered on the theme of diabetes increasing the risk for cardiovascular disease, with limited focus on why the specific model of diabetes was selected vs. other models.
Sex, age, strain, and sample size information	15 studies used only young male animals (only 6 reported females, 3 did not report sex studied, 1 did not report strain studied, and 1 did not report the age of the animals). Sample sizes were always clearly provided with majority of studies using an “*n*” of at least 5 or greater, and some studies reporting an “*n*” as high as 28.
Glucose homeostasis assessed	12 studies only provided data on fasting or ad libitum blood glucose and insulin levels (only 7 reported on additional indices of glucose homeostasis, e.g., glucose tolerance, insulin tolerance). A control group was included in several studies to demonstrate that the dietary intervention, genetic model, or STZ treatment exhibited the intended metabolic phenotype.
Cardiac physiology assessed	13 studies did not report on parameters of cardiac function (though this is not always a relevant end point to assess, e.g., studies whose primary goal is to study indices of atherosclerotic plaque formation or vessel function). When cardiac function was assessed, the primary method was ultrasound echocardiography, which frequently measured parameters of both systolic and diastolic function (9 studies reported on parameters of diastolic function), whereas invasive hemodynamics was also used in some studies.
***Recommendations to Improve Standardization***	
Provide a more robust rationale for choice of model and explain why a specific model of diabetes is selected vs. another (e.g., the study is addressing diabetic heart disease specifically and the development of reproducible diastolic dysfunction is necessary).	
Ensure accurate reporting of strain, sex, and age details. It is important to study both sexes, as there are sex-specific considerations regarding cardiac function and sex-specific considerations with glycemic status.	
More thorough investigations of the glycemic status of the animals should also be included to validate the model, especially if a pharmacotherapy is used.	
Details on the duration and composition of the control diet and the high-fat diet need to be reported.	
If status of diabetic heart disease is a major end point, both systolic and diastolic function should be measured.	

STZ, streptozotocin; ZDF, Zucker diabetic fatty.

It is important to note that models of diabetic heart disease are unique as they must model disrupted metabolism consistent with that observed in clinical diabetes in humans while also producing the cardiac dysfunction consistent with clinical phenotypes. The purpose for selecting a model of diabetes may relate to studying other mechanisms of diabetes and how they may impact the cardiovascular system (e.g., inflammation, oxidative stress, and microvascular function). However, our evaluation of these 24 studies relates to their utility in studying the pathology of diabetic heart disease.

Only approximately half of the 24 studies (11 to be exact) included in our evaluation measured parameters of cardiac function. Importantly, for the 11 studies that did report on parameters of cardiac function, the majority did include an assessment of both systolic and diastolic function. Of the 24 included studies, only seven reported on parameters of glycemia beyond simple measurement of fasting or ad-libitum blood glucose and insulin levels (e.g., glucose and/or insulin tolerance testing). The most frequently used model in our 24 included studies was the use of a high-fat diet to promote weight gain and ensuing obesity. However, as discussed previously, high-fat diet-induced obesity in rodents produces an insulin resistant, prediabetes phenotype, but not a true T2D phenotype while lacking notable cardiac dysfunction (56). There was also often variation in diet composition and duration for those studies that used a high-fat diet to promote weight gain and obesity, and it is necessary for researchers to provide these details for all diets (including the control diets) used in their studies. Other relevant concerns with the 24 included studies are the reliance on primarily young male animals (only 6 reported use of females), with multiple studies not reporting sex at all. It is readily apparent that the issues we observed in the 24 included studies in our evaluation of the current literature, published by the *American Journal of Physiology-Heart and Circulatory Physiology*, are persistent throughout the field. Therefore, our general recommendations are that researchers using these models need to provide more details regarding the rationale for their choice of diabetes model while providing sufficient details on the strain, age, and sex of the animals studied. Furthermore, the elevation of blood glucose levels does not definitively indicate diabetes, and additional indices of glucose homeostasis need to be reported (e.g., glucose tolerance and insulin tolerance testing). Finally, if an investigation is studying diabetic heart disease per se, regardless if it be the specific mechanisms involved or assessing a potential pharmacotherapy, it is critical that parameters of both systolic and diastolic function are measured. By applying these criteria, it is the hope of all authors of this review that the details provided herein will allow researchers to select the most relevant model to address their specific questions relating to the study of diabetic heart disease.

## MODELING ISCHEMIC INJURY AND HEART FAILURE IN DIABETES MODELS

Although many of the aforementioned dietary, genetic, and pharmacological-based models reproduce key features of diabetic heart disease, additional interventions are required to study myocardial infarction and heart failure in the context of diabetes. In this particular section, we will not describe in detail the methodologies behind the various experimental models of myocardial infarction and heart failure, which have already been extensively described in the following reviews (270–276). Instead, we will focus on their application to rodent models of prediabetes and diabetes, discussing in each case both their advantages and limitations.

To study acute changes in cardiac function in response to myocardial ischemia, the most frequently used techniques are the ex vivo isolated Langendorff and the isolated working heart perfusion methods. Ischemia is often implemented in these models by inducing a temporary global no-flow or low-flow ischemia for 20–30 min, following which one can assess contractility via LV-developed pressure and cardiac work/power upon reperfusion (270, 273). These approaches interrogate the response to ischemia in diabetes, but also facilitate therapeutic studies by prior treatment of T2D animals with compounds or acute administration of compounds into the perfusion apparatus. Despite diabetes increasing the risk for ischemic heart disease in humans, both poorer and improved cardiac adaptation to hemodynamic and ischemic stress have been reported in diabetic models, which may be due to variations in the severity of the diabetic phenotype and experimental conditions between studies (34, 117, 125, 151, 251, 277, 278). Isolated perfused heart studies in *ob*/*ob* mice, *db*/*db* mice, ZDF rats, mice/rats subjected to high-fat diet plus STZ-induced T2D, mice/rats with STZ-induced T1D, and Akita mice have consistently reported an elevation in myocardial fatty acid oxidation and corresponding decline in glucose oxidation (53, 95, 100, 111, 149, 215). Although increases in myocardial fatty acid oxidation are often thought to be detrimental to the pathology of diabetic cardiomyopathy, the addition of exogenous high levels of palmitate was reported to confer beneficial functional effects and improve redox balance in isolated working hearts from *db/db* mice under conditions of high glucose and isoprenaline stress (279). An advantage with isolated heart perfusion techniques is that they offer a highly controlled environment for one to manipulate the substrate and hormonal concentrations to which the heart is exposed. A limitation is that the metabolic milieu the heart sees in diabetes is vastly different from that of a healthy heart. However, for comparative purposes, substrate and hormonal levels are kept identical between hearts from lean versus diabetic animals. It is also important to acknowledge that these ex vivo perfusion modalities are not equivalent to in vivo measurement of cardiac function, but allow interrogation of different research questions.

To study diabetes-related myocardial infarction and heart failure, surgical procedures involving either ligation of the left anterior descending (LAD) coronary artery or transverse aortic constriction (TAC), respectively, are used with the various animal models of diabetes described earlier. Although both surgical models have been validated with their own strengths and limitations (53, 95, 100, 111, 149, 215), there are several factors one must consider when choosing which diabetes model to use in tandem. For example, halogenated anesthetics should be avoided during temporary ligation of the LAD coronary artery to model acute myocardial infarction, because of their cardioprotective effect in nondiabetic animals, which have been reported to be attenuated with diabetes (280, 281). In addition, variations in experimental parameters such as duration of the diabetic state, changes in circulating insulin concentrations, but also differences in dietary composition (e.g., dietary fat content) can markedly influence outcomes, with infarct sizes in the animal models described earlier reported to be larger, smaller, or similar to that of nondiabetic controls (282–284). Regarding TAC surgery, although most studies demonstrate an exacerbated cardiac hypertrophic response in the setting of diabetes, a major criticism is that the rapid increase in afterload created by the surgery more closely mimics aortic stenosis than the progressive rise in blood pressure associated with diabetes. Because of the relative early development of obesity and insulin resistance, LAD coronary artery ligation or TAC is often performed using *db*/*db* mice as the animal model of choice. In line with diabetes worsening myocardial infarction outcomes in patients, *db*/*db* mice exhibit a worsening of systolic function versus their nondiabetic controls in response to a 45-min LAD coronary artery occlusion followed by 28 days of reperfusion (285). Conversely, *db*/*db* mice demonstrate robust protection against TAC-induced HFrEF (115). Because the failing heart is often thought to be energy-starved and characterized by reduced fatty acid oxidation rates (286), it has been proposed that the marked elevation in myocardial fatty acid oxidation in *db*/*db* mice is responsible for their cardioprotection against TAC (115). Similarly, mice with T2D in response to a high-fat diet plus low-dose STZ do not develop systolic dysfunction following TAC surgery (287), which may also be related to T2D-mediated increases in myocardial fatty acid oxidation (95, 100). Despite T1D also being associated with increased risk for heart failure (288), mice subjected to STZ-induced T1D are also protected against TAC-induced heart failure, as the decline in systolic function and ensuing cardiac hypertrophy are not as robust as that observed in their nondiabetic counterparts (289).

In general, a major limitation with the vast majority of LAD coronary artery ligation or TAC studies in animal models of diabetes is that both female mice and aged mice are often overlooked. Furthermore, many of the aforementioned animal models of diabetes have not been extensively validated regarding their response to LAD coronary artery ligation or TAC. A reason for the lack of validation relates to the increased cost, time, and expertise involved with performing surgical interventions in animal models of diabetes, which necessitates the requirement of additional control groups. Thus, investigators will frequently not include low-fat diet-fed lean control groups and simply study cardiovascular outcomes in diabetic animals following either sham surgery or LAD coronary artery ligation/TAC surgery, or in response to vehicle control versus pharmacological intervention. Nonetheless, because of the growing appreciation of monitoring cardiovascular outcomes in diabetes, it is our hope that the next decade will lead to much-needed advancement of knowledge in this area.

## LARGE ANIMAL MODELS OF DIABETES

In general, mouse models of diabetes are more frequently used to study diabetic heart disease because of their overall low-cost, ease of use due to development of specialized equipment, and ease of genetic manipulation. Nonetheless, they do have their own limitations as a model species to study, which from a diabetes standpoint was nicely highlighted in a recent study by de Cabo and coworkers (290). They observed in comparisons of >1,000 mice from the Study of Longitudinal Aging in Mice, >250 nonhuman primates (NHPs) from the National Institute of Aging and the Wisconsin National Primate Research Center, and >3,000 humans from the Baltimore Longitudinal Study of Aging, that aging-related increases in fasting plasma glucose correlate positively with risk for mortality in both NHPs and humans. In contrast, aging is associated with a reduction in fasting plasma glucose in mice. Moreover, numerous novel pharmacotherapies that show promise for the treatment of obesity and/or T2D in rodents often fail to translate to humans. Hence, it is imperative that pharmacological effectiveness and safety/toxicology also be assessed in larger animal models.

The use of NHPs and swine models have proven useful for the study of obesity and diabetes (both T1D and T2D). Rhesus and Cynomolgus macaques can be susceptible to spontaneous obesity that progresses to insulin resistance (291, 292), whereas dietary manipulations in macaques, similar to what has been described in rodents, can also be used to induce obesity and insulin resistance (293). With regard to swine, minipig species (Ossabaw, Gottingen, and Yucatan) are frequently used to study obesity/T2D in response to dietary manipulations, where they can be maintained into adulthood at somewhat reasonable costs while also being amenable to genetic manipulation (294). STZ and alloxan can also be used to induce T1D in the aforementioned models, though because of variability of response to these chemicals, surgical methods to induce partial or complete pancreatectomy have also been used (294). Despite these larger animal models having strong translational relevance to that of humans, there has been surprisingly minimal exploration of in vivo cardiac function in obese and/or diabetic NHPs and swine. Accordingly, how accurately these larger animal models recapitulate key features of diabetic heart disease (e.g., early diastolic dysfunction seen in diabetic cardiomyopathy) is underexplored.

## FINAL SUMMARY AND IMPORTANT CONSIDERATIONS FOR THE FUTURE

A wide variety of models to study both the pathology, as well as the development of new therapies for both T1D and T2D, have been developed, each of which comes with their own unique set of advantages and disadvantages (summarized in Figs. 1–4). Due to the growing recognition of the importance of also managing cardiovascular disease in diabetes, it is imperative that we also validate these models of diabetes on their ability to reproduce features of diabetic heart disease in humans. Furthermore, since these models for most part do not result in atherosclerosis, myocardial infarction, and heart failure, it also necessitates validating how responsive they are to surgical models of these macrovascular cardiovascular diseases. One notable example highlighting the importance of this issue is seen with *db*/*db* mice, which appear to exhibit diastolic dysfunction in a multitude of studies, indicating that they are a valid model for studying diabetic cardiomyopathy in humans. On the contrary, due to their resistance to TAC-mediated impairments in systolic function, they are not an ideal model to study T2D-related HFrEF.

Although some models of diabetes have interrogated sex-specific differences with regard to their ability to produce diabetic heart disease, it is imperative that this be more extensively characterized in all models, considering that diabetic cardiomyopathy is more prevalent in women (295). For models where cardiac dysfunction is absent in female animals, an important aspect to consider relates to housing temperature. The vast majority of referenced literature in this review encompasses mice studied at room temperature, but thermoneutrality for mice is between 27°C and 30°C. Of relevance, female mice are highly resistant to obesity-induced nonalcoholic fatty liver disease and subsequent steatohepatitis, but this resistance is extinguished if the mice are housed at thermoneutrality (296). More recently, studies in male mice housed at either room temperature or thermoneutrality have demonstrated that thermoneutrality lowers heart rate and mean arterial pressures in lean mice, whereas these actions are blunted in mice subjected to high-fat diet-induced obesity (252). Taken together, as the field continues to collaborate and refine the current models of diabetes, while also developing new models, this should result in further improvements in our ability to clinically manage diabetes-related cardiovascular disease.

## GRANTS

L. C. Heather is a British Heart Foundation Intermediate Basic Science Fellow Grant FS/17/58/33072. G. V. Halade is supported by the National Institutes of Health (NIH) Grants R01 HL132989 and R01 HL144788. R. Harmancey is supported by NIH Grants R01 HL136438, P20 GM104357, and P01 HL051971. K. M. Mellor is supported by the New Zealand Marsden Fund Grant 19-UOA-268 and the Health Research Council of New Zealand Grant 19/190. P. K. Mishra is supported by NIH Grant P20 GM104320 of the Nebraska Center for the Prevention of Obesity Diseases. E. E. Mulvihill is supported by Canadian Institutes of Health Research (CIHR) Project Grant PJT-156136 and a New Investigator Award from the Heart and Stroke Foundation of Canada. M. Nabben is supported by a Dutch Heart Foundation Dekker Senior Scientist Grant 2019T041. M. Nakamura is supported by NIH Grant R01 HL155766. M. Ruiz is an FRQS Junior 1 Research Scholar under Grant 281577. A. R. Wende is supported by NIH Grant R01 HL133011. J. R. Ussher is supported by CIHR Project Grant PJT-159648 and a Tier 2 Canada Research Chair (Pharmacotherapy of Energy Metabolism in Obesity).

## DISCLOSURES

No conflicts of interest, financial or otherwise, are declared by the authors.

## AUTHOR CONTRIBUTIONS

L.C.H., A.D.H., G.V.H., R.H., K.M.M., P.K.M., E.E.M., M.N., M.N., O.J.R., M.R., A.R.W., and J.R.U. drafted manuscript; L.C.H., A.D.H., G.V.H., R.H., K.M.M., P.K.M., E.E.M., M.N., M.N., O.J.R., M.R., A.R.W., and J.R.U., edited and revised manuscript; L.C.H., A.D.H., G.V.H., R.H., K.M.M., P.K.M., E.E.M., M.N. M.N., O.J.R., M.R., A.R.W., and J.R.U. approved final version of manuscript.
